# Nutrient management and medium reuse for cultivation of a cyanobacterial consortium at high pH and alkalinity

**DOI:** 10.3389/fbioe.2022.942771

**Published:** 2022-08-11

**Authors:** Alexandre J. Paquette, Agasteswar Vadlamani, Cigdem Demirkaya, Marc Strous, Hector De la Hoz Siegler

**Affiliations:** ^1^ Department of Geoscience, University of Calgary, Calgary, AB, Canada; ^2^ Department of Chemical and Petroleum Engineering, University of Calgary, Calgary, AB, Canada

**Keywords:** nutrient management, Candidatus “Phormidium alkaliphilum”, nutrient uptake, microalgae cultivation, reuse, spent medium

## Abstract

Alkaliphilic cyanobacteria have gained significant interest due to their robustness, high productivity, and ability to convert CO_2_ into bioenergy and other high value products. Effective nutrient management, such as re-use of spent medium, will be essential to realize sustainable applications with minimal environmental impacts. In this study, we determined the solubility and uptake of nutrients by an alkaliphilic cyanobacterial consortium grown at high pH and alkalinity. Except for Mg, Ca, Co, and Fe, all nutrients are in fully soluble form. The cyanobacterial consortium grew well without any inhibition and an overall productivity of 0.15 g L^−1^ d^−1^ (AFDW) was achieved. Quantification of nutrient uptake during growth resulted in the empirical formula CH_1.81_N_0.17_O_0.20_P_0.013_S_0.009_ for the consortium biomass. We showed that spent medium can be reused for at least five growth/harvest cycles. After an adaptation period, the cyanobacterial consortium fully acclimatized to the spent medium, resulting in complete restoration of biomass productivity.

## Introduction

Photosynthetic microorganisms such as cyanobacteria and eukaryotic microalgae have been proposed as a source of biomass for the production of bioenergy and bioproducts (Chisti, 2007; Mata et al., 2010; Khan et al., 2018). These microorganisms can grow on non-arable land and can potentially reach higher areal productivities than traditional food crops (Chisti, 2007; Brennan, 2010; Johnson and Wen, 2010; Christenson and Sims, 2012; Singh et al., 2017). While eukaryotic microalgae are investigated for their high lipid content, possibly contributing to renewable energy, cyanobacteria are mainly cultivated at large scale to produce high value products such as pigments, proteins, and vitamins (Chisti, 2007; Hoh et al., 2016; Singh et al., 2017).

Current large-scale systems for algal cultivation have a high-water footprint and nutrient demand (Scott et al., 2010; Zhang et al., 2014; Farooq et al., 2015; Lu et al., 2020). In fact, some studies have reported that to produce 1 L of microalgal biodiesel, over 3000 L of water is required (Farooq et al., 2015). Nutrient demand is typically associated with nitrogen and phosphorus, as they are energy intensive to obtain or their worldwide reserves are depleting (Rösch et al., 2012). Although carbon dioxide is less associated with the topic of nutrient demand, it is also crucial for algal biomass production and its supply to the culture needs to be considered. Depending on the growth conditions carbon can be supplied as CO_2_ that is bubbled into the media or in the form of bicarbonate (HCO_3_^−^) (Chi et al., 2011; Rafa et al., 2021; Zhu et al., 2021). For systems that bubble in CO_2_ there are high capital and operating costs associated with CO_2_ transportation and bubbling, as well as high energy requirements (Chi et al., 2011; Rafa et al., 2021; Zhu et al., 2021). In systems that use bicarbonate there are costs associated with the high concentration of bicarbonate in the growth medium. Some studies have shown that the use of bicarbonate may also avoid costs by preventing culture crashes (Rafa et al., 2021; Zhu et al., 2021). As the global demand for high value cyanobacterial products increases (Singh et al., 2017; Bhalamurugan et al., 2018), the water and nutrient requirements will only increase, potentially leading to an unsustainable process (Hannon et al., 2010; Farooq et al., 2015; Lu et al., 2020).

To implement an effective cultivation strategy and mitigate the high water and nutrient demand, multiple strategies have been proposed: *1*) usage of nutrient-rich wastewater streams as a main source of nutrients and *2*) re-use of spent media with supplementation of the depleted nutrients (Acién Fernández et al., 2012; Acién Fernández et al., 2018; Barbera et al., 2018). Although mitigation with nutrient-rich wastewater streams is a promising strategy, it could potentially introduce foreign substances (e.g., heavy metal ions, pathogenic microbes, *etc*.) into the cultivation system. Therefore, it might not be a viable option for biomass that is cultivated for food and pharmaceuticals (Barbera et al., 2018).

Cultivation of alkaliphilic, “high pH loving,” cyanobacteria has gained interest over the recent years because of their robustness and ability to capture carbon dioxide directly from the atmosphere (Bell et al., 2016; Canon-Rubio, 2016; Sharp et al., 2017; Zhu et al., 2018; Ataeian et al., 2019; Chowdhury et al., 2019; Kim et al., 2019; Berthold et al., 2020). Previously, we enriched a consortium consisting of the alkaliphilic filamentous cyanobacterium *Candidatus* “Phormidium alkaliphilum” (80–90%) and associated heterotrophs (Ataeian et al., 2021; Ataeian et al., 2022). It was enriched from soda lakes located on the Cariboo Plateau (British Columbia, Canada) and was cultivated at a pH of up to 11.2 and with 0.5 mol/L of combined carbonates. This pH was high enough to demonstrate regeneration of spent media with carbon dioxide captured directly from the air (Ataeian et al., 2019). Long-term, crash-free productivity was shown in the laboratory (15.2 ± 1.0 g L-1 d-1, Ataeian et al. (2019)), as well as in an outdoor, high latitude pilot plant (5.8 g/m^2^/d, Haines et al. (2022)). The consortium contains 11–17% phycocyanin (Ataeian et al., 2021), which is a valuable, nutritious, and healthy blue pigment. Consortium composition at harvesting contains 60.9% protein, 13.4% lipids and 12% carbohydrates (Sharp et al., 2017; Demirkaya et al., 2022). Both productivity and biomass composition were typical of cyanobacteria, including *Arthrospira* (*Spirulina*). Although replenishing of inorganic carbon using air was a major step forward, reuse of other nutrients (P, N, Mg, S, Na, K, Ca, and Fe) is as important to make the cultivation system sustainable.

Using wastewater as a source of nutrients would not work for such a high-alkalinity cultivation system, because if an alkaline medium is combined with wastewater for cultivation, the alkalinity would be lost by dilution with non-alkaline wastewater. Re-use of spent media would mitigate both water and nutrient demand (Farooq et al., 2015; Lu et al., 2020). One study found that reusing spent media can reduce nutrient and water usage by 55 and 84%, respectively (Yang et al., 2011). The biggest advantage to reusing spent media is the cost savings on water, water pumping and nutrients (Yang et al., 2011; Farooq et al., 2015; Lu et al., 2020). Therefore, it is important to understand the effect of spent medium on biomass growth. Re-use of spent medium has been shown to increase, decrease or have no effect on biomass growth, depending on many factors such as culture conditions and harvesting methods (Lu et al., 2020). One area where the reuse of spent medium has not been well studied for biomass growth is in the cultivation of alkaliphilic cyanobacteria.

Here, we extend our previous work on the cultivation of an alkaliphilic cyanobacterial consortium and describe the nutrient solubility/availability and nutrient uptake rates at high pH (>10) and alkalinity (0.5 M). We also design and demonstrate cultivation conditions that allow re-use of the spent growth medium. The objectives of this study are to *1*) determine the solubility of the provided nutrients at high pH, *2*) investigate which nutrients are present at the end of a cultivation cycle and *3*) determine if the alkaliphilic cyanobacterial consortium can be grown with recycled, spent media.

## Materials and methods

### Cultivation conditions

A laboratory grown microbial consortium dominated by cyanobacteria was used in all experiments (Ataeian et al., 2019; Ataeian et al., 2021; Ataeian et al., 2022). This consortium was originally enriched from photosynthetic microbial mats obtained from soda lakes located on the Cariboo Plateau (British Columbia, Canada) (Sharp et al., 2017). A synthetic growth medium was formulated to simulate the high pH and alkalinity conditions of the soda lakes (Vadlamani et al., 2017; Ataeian et al., 2019). This medium, also used here, contained the following: Na_2_CO_3_ (210.98 mM), NaHCO_3_ (77.85 mM), NaNO_3_ (3.06 mM), NH_4_Cl (0.92 mM), KH_2_PO_4_, (1.44 mM), MgSO_4_·7H_2_O (1 mM), CaCl_2_·2H_2_O (0.17 mM), NaCl (0.43 mM), KCl (6.04 mM) FeCl_3_·6H_2_O (0.04 mM) and 300 μl/L of a trace metal solution. The trace metal solution contained H_3_BO_3_ (9.7 mM), MnCl_2_·4H_2_O (1.26 mM), ZnCl_2_ anhydrous (0.15 mM), CuCl_2_·2H_2_O (0.11 mM), Na₂MoO₄·2H_₂_O (0.07 mM), CoCl₂·6H₂O (0.06 mM), NiCl_2_·6H_2_O (0.04 mM), KBr (0.08 mM).

Initial experiments to assess the nutrient uptake during growth was carried out in 1 L Erlenmeyer flasks, filled with 0.5 L of medium, for 4 days. In the second set of experiments, spent media was used for cultivation and the experiments were carried out in 12 L carboys for a growth period of 6 days. Both experiments were carried out in triplicates and full spectrum LED lights (T5H0, 6400K, Sunblaster Holdings ULC, Canada) were used to provide a light intensity of 200 µmol photons·m^−2^s^−1^, operating with a light:dark cycle of 16:8 h (Ataeian et al., 2019). In both experiments the cyanobacterial consortium was cultivated as suspended cells and suspension cultures were continuously agitated using magnetic stir plates operating at 340 rpm.

### Analytical methods

#### Supernatant analysis

The pH of the culture samples obtained during the incubation was measured using a pH meter (Seven Compact^™^ S220, Mettler Toledo, United States). The cultures were then centrifuged for 10 min at 3,900 *g* (Allegra X-22R, Beckman Coulter, United States). The supernatant obtained after centrifugation was analysed for total alkalinity (TA), anions, cations, and total nitrogen concentrations. In brief, TA was measured using a G20 compact titrator (Mettler Toledo, United States); 40 ml of supernatant was taken in a beaker and titrated with 0.2N H_2_SO_4_ until the samples reached an endpoint of pH = 4.3 (Vadlamani et al., 2019). Bicarbonate and carbonate concentrations were calculated using the measured pH and total alkalinity (TA) (Vadlamani et al., 2017; Vadlamani et al., 2019).

The nitrate concentration was measured using an ion chromatograph equipped with an IonPac AS18 anion and a conductivity detector (DIONEX ICS 2000; Thermo Fisher, United States) (Vadlamani et al., 2017; Vadlamani et al., 2019). The ammonium concentration was determined colorimetrically as previously described (Sims et al., 1995). The total nitrogen concentration was assessed using a scaled down version of the Persulfate Digestion method (Hach Method 10071, Hach, United States) (Vadlamani et al., 2017; Vadlamani et al., 2019).

#### Quantification of cations

Sodium, potassium, phosphorus, sulfur, magnesium, calcium, and iron were analyzed in both freshly prepared medium, and supernatant obtained after growth. All measurements were performed using an Agilent 8800 Triple Quadrupole Inductively Coupled Plasma Mass Spectrometer (ICP-QQQ, Agilent Technologies, Tokyo, Japan). The ICP-QQQ was equipped with an SPS4 autosampler, which uptakes 3 ml of sample for analysis. Fresh media and culture samples obtained after each day of incubation were first centrifuged at 4500 rpm for 5 min to spin down cells. Next, the supernatant was split into two fractions. To one fraction, 5% HNO_3_ was added, reducing the pH to less than 3 and dissolving any precipitated cations. The pH of the other fraction was not adjusted. By comparing the mineral content of these two fractions, the difference between the dissolved and total amounts could be determined for each ion (Hanifzadeh et al., 2018). Immediately before ICP analysis, samples were filtered through 0.2 µm mixed cellulose esters (MCE) membrane filters, to remove any remaining solids. The cation concentrations for both the fresh medium and supernatant were estimated from calibration curves generated by applying the same protocols to the standards. The relative standard deviation of ICP-QQQ measurements was within 4%.

#### X-ray mapping of precipitates

To determine the chemical nature of precipitates in the growth medium, an FEI Quanta FEG 250 environmental field emission scanning electron microscope (E-FESEM) in combination with a Bruker QUANTAX Energy Dispersive X-ray Spectroscopy (EDS) system was used. In brief, a freshly prepared medium was filtered using 0.2 µm mixed cellulose esters (MCE) membrane filter to separate the precipitates. Once all the media had passed through the filter, Mili-Q water was used to wash the filter. After washing the filter membrane two times, the filter was air dried for 24 h. A small portion of the dried filter membrane was mounted onto a stub using double sided carbon tape without any surface modification. The stub containing the filter membrane was then analyzed to obtain the elemental maps using the E-FESEM/EDS system (operated at primary energy – 15 KeV). These elemental maps were used to infer the chemical composition of the precipitates.

#### Visual MINTEQ modelling

Visual MINTEQ was used to estimate ion speciation in the high pH, alkaline medium. The chemical equilibrium model of Visual Minteq 3.1 (https://vminteq.lwr.kth.se/) is based on the program PC MINTEQA2 (Allison et al., 1991; Gustafsson, 2011). Computations were carried out using initial experimental conditions: *1*) pH (10.46), *2*) temperature (20°C), *3*) Ion concentration and *4*) Ionic strength (0.73 M) as input variables. With Visual Minteq we estimated, for each element: *1*) the concentration of dissolved ions(s); *2*) the fraction bound to ligands and *3*) the fraction precipitated (Gustafsson, 2011).

#### Biomass productivity and elemental analysis

The wet biomass obtained after centrifugation was frozen at −80°C overnight and then freeze dried at −50°C at a pressure of 1 mPa using a bench top freeze dryer (Labconco, Kansas City, MO, United States) (Vadlamani et al., 2019). Ash content of the freeze-dried biomass was then analysed with a muffle oven as previously described (National Renewable Energy Laboratories, NREL, Sluiter et al., 2008). The ash content was used to estimate ash-free biomass concentrations and productivity using the equations provided in the Supplementary Information.

Carbon, nitrogen, and hydrogen content of the freeze-dried biomass was determined as previously described (Ataeian et al., 2019). Other elements (K, P, S, Mg, Ca, and Fe) in the biomass were determined by ICP-MS analysis of the digested biomass. First, digestion of the biomass was carried out using a MARS 6 – Microwave Digestion System (CEM Corporation, United States) (Hanifzadeh et al., 2018). In brief, 10 ml of HNO_3_ were added to about 50 mg of freeze-dried biomass and digested at 250°C and at a pressure of 55.16 bar for 30 min. These samples were then diluted with Milli-Q water (to fit in the calibration range) and analyzed for metallic (K, Mg, Ca, Fe) and some non-metallic (P, and S) elements using an ICP-MS (ICP-MS, Xseries 2, Thermo Scientific, United States) (Hanifzadeh et al., 2018). The measured elemental concentrations were then used to estimate nutrient uptake rates.

Carbohydrate content was analyzed by the sulfuric acid-phenol method; briefly, 5 mg of freeze-dried biomass was resuspended into 2 ml of DI water and then ultrasonicated using a 50 W Ultrasonic Homogenizer with a ⅛ tip (VWR, United States) for 10 min 200 µl of the ultrasonicated biomass were transferred into a 2 ml tube and 200 µl of 5% phenol and 1 ml of concentrated sulfuric acid were added. The tubes were incubated for 30 min at room temperature and then the absorbance was measured at 450 nm using a SpectraMax iD3 Multi-Mode Microplate Reader (Molecular Devices, San Jose, California, United States). The carbohydrate contents were estimated from a calibration curve generated by applying the same protocols to glucose standards (Masuko et al., 2005).

Protein content was measured following a modified Lowry assay as described by Slocombe et al. (2013). Briefly, 5 mg of freeze-dried biomass were added to 200 µl of 24% (v/v) trichloroacetic acid and incubated at 95°C for 15 min. After cooling to room temperature, 600 µl of water were added and tubes centrifuged at 15,000 *g* for 20 min at 4°C. The pellet was resuspended using 0.5 ml of Lowry Reagent D (24:0.5:0.5 ratio solution of Lowry reagent A (2% w/v of anhydrous Na_2_CO_3_ in 0.1 N NaOH), reagent B (1% w/v NaK tartrate tetrahydrate), and reagent C (0.5% w/v CuSO_4_⋅5H_2_O in H_2_O)) and then incubated for 2 h at 55°C. After incubation the samples were cooled to room temperature and centrifuged at 15,000 *g* for 20 min. The supernatant was then transferred to new tubes and the pellet was discarded. For quantification, 950 µl of Lowry Reagent D were added to 50 µl of the protein extract, mixed quickly by inversion, and incubated at room temperature for 10 min. Finally, 100 µl of diluted Folin-Ciocalteu phenol reagent (2N, Sigma-Aldrich, United States) were added, vortexed, and incubated at room temperature for 30 min (Slocombe et al., 2013). The absorbance was measured at 600 nm using a SpectraMax iD3 Multi-Mode Microplate Reader (Molecular Devices, United States). The protein content was estimated from a calibration curve generated by applying the same protocol to bovine serum albumin standards, prepared from a 30% solution (VWR, United States).

#### Statistical analysis

One-way analysis of variance (ANOVA) was conducted to identify significant differences between nutrient concentrations. In each case, a *p* value less than 0.05 was considered significant with a sample size, *n* = 3. Statistical analyses were performed using MS Excel.

## Results and discussion

### Nutrient solubility

Although it is hard to know to what extent an element is “bio-available,” at least we can be fairly confident that elements dissolved as ions in the medium are available for uptake by microorganisms (Suzuki et al., 1995; Lee et al., 2009). Because of the high pH (>10.4) and high ionic strength (0.73 M) of the growth medium, some elements (especially Mg, Ca, and Fe) were likely to precipitate (Vandamme et al., 2012). We performed a comprehensive analysis on the freshly prepared medium to determine the solubility of each element.

**FIGURE 1:**
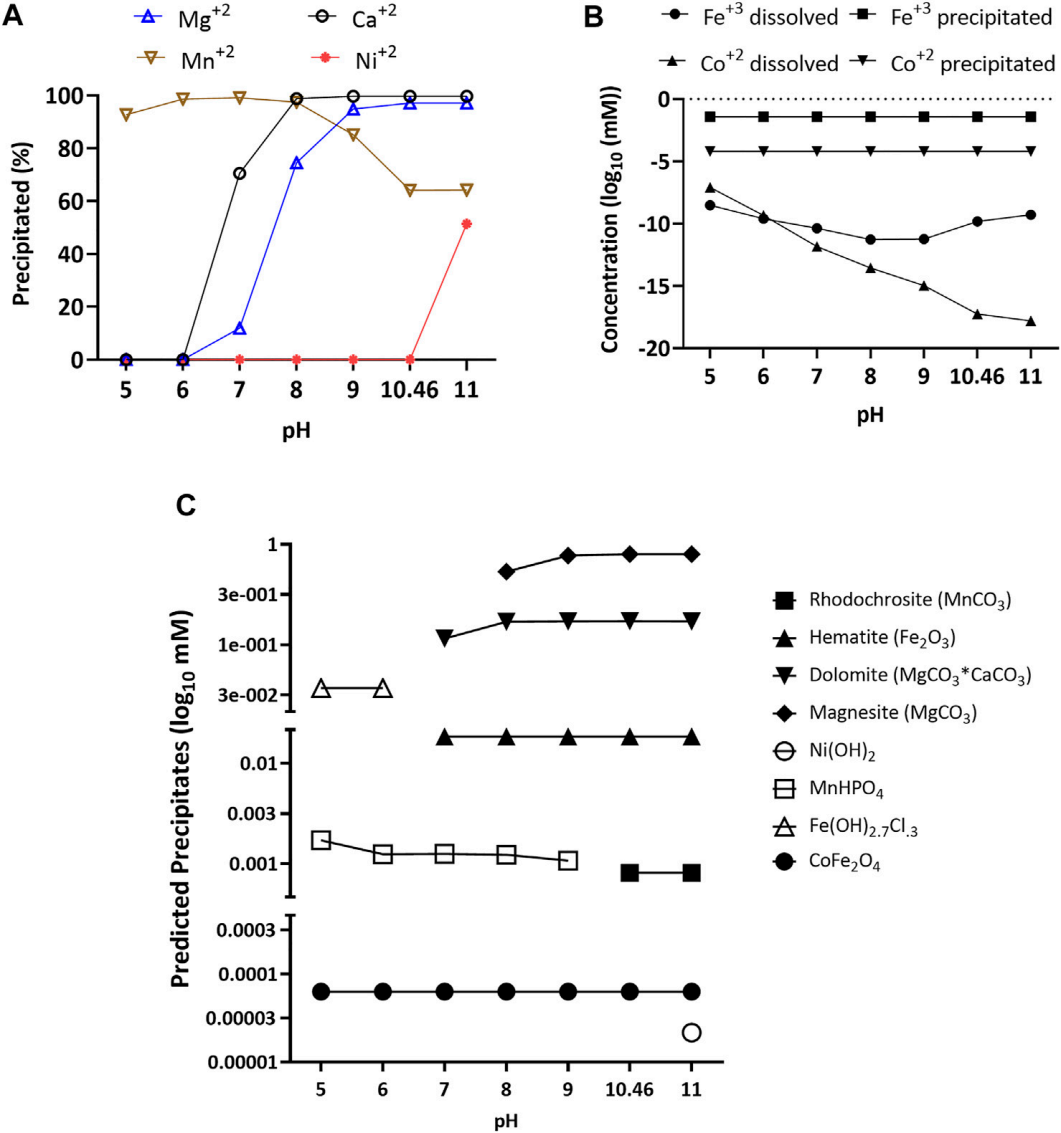
Percentage of magnesium, calcium, nickel, and manganese precipitated **(A)**, concentration dissolved and precipitated for iron and cobalt **(B)**, and predicted precipitates **(C)** of calcium, magnesium, cobalt, nickel, and iron over a pH range of 5–11 with an ionic strength of 0.73 M. Data was obtained using Visual Minteq 3.1 equilibrium model.

First, we used Visual Minteq 3.1 software (KTH, Sweden) to predict both the likelihood of precipitation and the nature of expected precipitates in the high alkalinity medium as a function of pH. For Mg^2+^, Ca^2+^, and especially Fe^3+^ and Co^2+^, the salts added to the medium were expected to precipitate nearly completely at the actual medium pH of 10.46 (Figures 1A,B). For, Mn^2+^, 64% of the added manganese was expected to precipitate. Lastly, nickel was only predicted to precipitate above pH 11. Further, under equilibrium conditions, the software predicted that above pH 7, both Mg and Ca would form carbonate salts such as dolomite (MgCO_3_*CaCO_3_) and magnesite (MgCO_3_) (Figure 1C). For iron, it would mainly precipitate as hematite (Fe_2_O_3_, Figure 1C), with production of a minor fraction of cobalt ferrite (CoFe_2_O_4_, Figure 1C). Manganese would mainly precipitate as rhodochrosite (MnCO_3_) from pH 10.46 to 11 and as MnHPO_4_ from pH 5 to 9. Lastly, nickel at pH 11 would precipitate as Ni(OH_2_).

**FIGURE 2:**
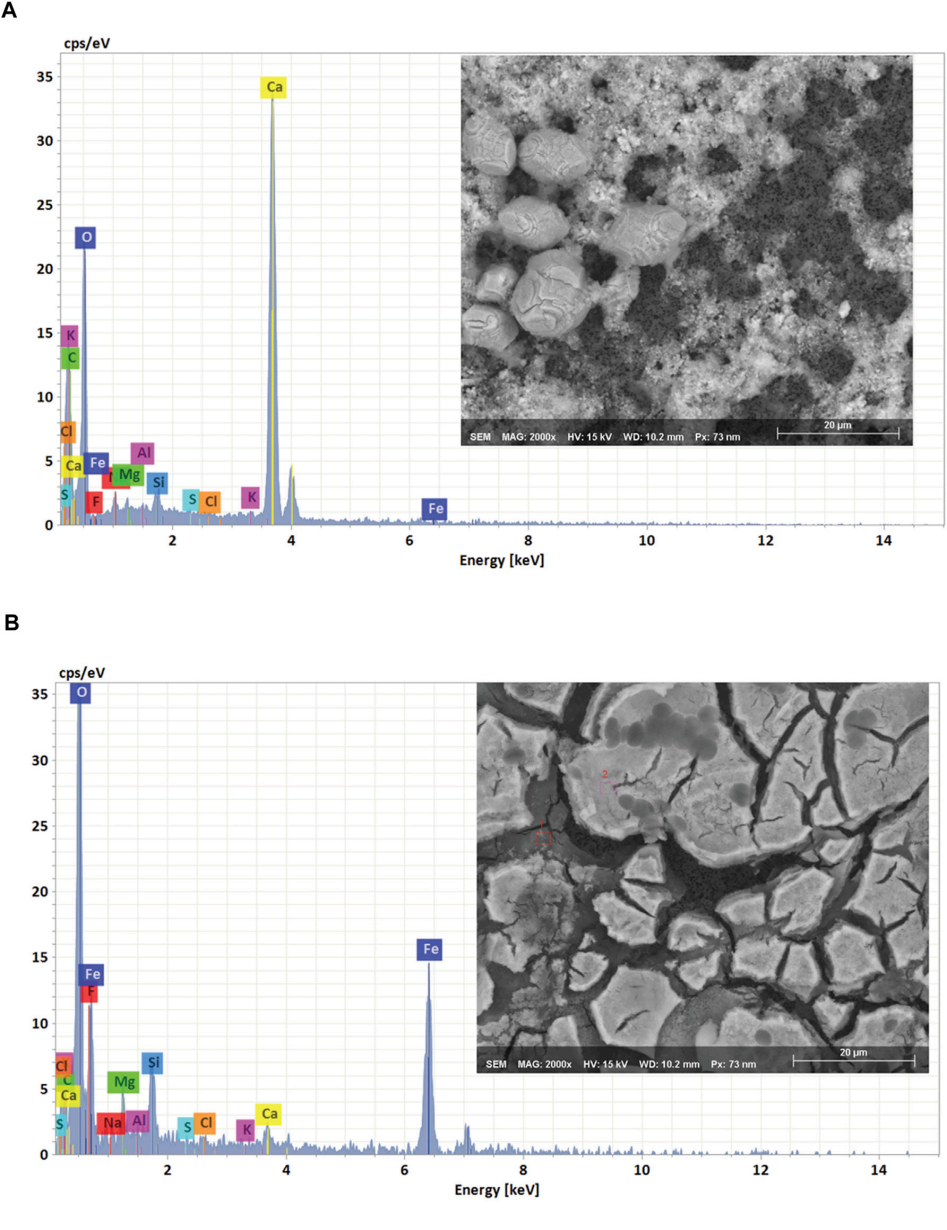
SEM image and EDS spectrum of the recovered precipitates **(A)** CaCO_3_ and **(B)** Fe (OH)_3_.

To verify the equilibrium model’s predictions, we collected precipitates from freshly prepared medium and analyzed them using a scanning electron microscope with energy-dispersive X-ray spectroscopy (SEM-EDS). The SEM images shown in Figures 2A,B indicate that the surface of the filter was covered with amorphous precipitates. EDS analysis confirmed experimentally that the amorphous precipitates mainly consisted of calcium carbonate and iron oxide (Figures 2A,B). For Mn, Co, and Mg it was difficult to identify the nature of the precipitates by using the EDS analysis, because the signals for Ca and Fe overpowered the spectrum. It was also possible that the precipitates formed from Mn, Co and Mg passed through the 0.2 µm filter and for that reason weren’t observed in the EDS analysis.

The fresh culture medium at pH = 10.46 was further analyzed using inductively coupled plasma mass spectrometry (ICP-MS) to determine the concentrations of dissolved sodium, potassium, phosphorus, sulfur, magnesium calcium, and iron. In parallel, the pH of the culture medium was decreased to less than 3, to dissolve any precipitated minerals, and analyzed it on ICP-MS. Together, these two measurements provided the dissolved and total amounts for each element, respectively. The ICP-MS analyses were compared with the Minteq predictions, and the amounts actually added to the growth medium (Table 1). ICP-MS showed that the added sodium, potassium, phosphate, and sulfate remained fully dissolved (Table 1). ICP-MS also showed that this was not the case for magnesium, calcium, and iron, indicating significant precipitation (ANOVA single factor, *p* = 0.02, Table 1). The experimentally determined concentration of dissolved Mg, Ca and Fe was still higher than the Minteq predictions. This indicated that the high pH culture medium was supersaturated in Mg, Ca, and Fe. Supersaturation of carbonate salts is a well-known phenomenon occurring in many natural waters (Minde et al., 2020). Despite supersaturation, the soluble fraction of Mg, Ca and Fe was significantly reduced due to precipitation in the high pH medium.

**TABLE 1:** Concentration (mM) of nutrients at high pH (10.4), reduced pH (<3), and solubility (%).

Elements	Expected concentration (mM)	Concentration in solution (mM)		Solubility (%)
		Analyzed at pH 10.4^a^	Analyzed at pH < 3^b^	
C-HCO_3_^−^ ^c^	77.85	33.3 ± 0.8	N/A	N/A
N-NO_3_^−^ ^d^	3.06	2.97 ± 0.07		100
N-NH_4_^+^ ^e^	0.92	0.75 ± 0.04		82
Na	500	483.6 ± 5.5	476.5 ± 26.8	100
Mg	1	0.3 ± 0.04	1.0 ± 0.09	30
K	8.5	7.96 ± 0.42	8.0 ± 0.74	100
P	1.44	1.44 ± 0.05	1.4 ± 0.1	100
S	1	1.0 ± 0.07	0.88 ± 0.08	100
Ca	0.25	0.08 ± 0.02	0.25 ± 0.01	32
Fe	0.04	0.007 ± 0.001	0.02 ± 0.001	17

a To determine the total amounts of elements by ICP-MS, the pH of growth medium was reduced using 5% HNO_3_.

b High pH growth medium was directly analyzed on ICP-MS to obtain the concentration of dissolved elements.

c (bi)carbonate was calculated using TA which was determined by titration with 0.2 H_2_SO_4_, and pH values.

d Nitrate was measured using an ion chromatograph.

e Ammonium concentrations were determined using colorimetry.

For inorganic carbon, it is well known that at pH 9–10, the dissolved CO_2_ concentration is low and HCO_3_^−^ is the dominant species. As the pH further increases (pH > 10), CO_3_^2−^ becomes dominant. Since cyanobacteria have the ability to utilize both CO_2_ and HCO_3_^−^, but not CO_3_^2−^ (Raven, 1994), it was important to determine the bicarbonate concentration in the high pH medium. The actual HCO_3_^−^ concentration was calculated using the measured total alkalinity (TA, 0.5 ± 0.003 M) and pH (10.46 ± 0.02) values. The actual HCO_3_^−^ concentration (33.3 ± 0.8 mM) was lower than the amount of bicarbonate added to the growth medium (77.9 mM, Table 1). This was caused by equilibration (outgassing) of dissolved CO_2_ with ambient air, increasing pH and leading to the production of CO_3_^2−^ (Wolf-Gladrow et al., 2007).

Nitrogen, another important nutrient, was also analysed for solubility. We added 3.98 mM total N to the medium, in the form of NaNO_3_ (3.06 mM) and NH_4_Cl (0.92 mM). Nitrate measurements indicated that close to 100% of the added NO_3_^−^ remained dissolved in the medium (2.97 ± 0.07 mM, Table 1). On the other hand, measured NH_4_^+^ concentrations indicated that the medium contained 20% less ammonium than was added (0.75 ± 0.04 mM compared to 0.92 mM, Table 1). The decrease in the N-NH_4_^+^ could be explained by outgassing of volatile NH_3_ from the medium at high pH (Körner et al., 2001). Overall, more than 90% (3.72 mM) of the nitrogen added remained in the media to support cyanobacterial growth.

### Biomass growth and nutrient uptake

#### Growth profile of cyanobacterial consortium

**FIGURE 3:**
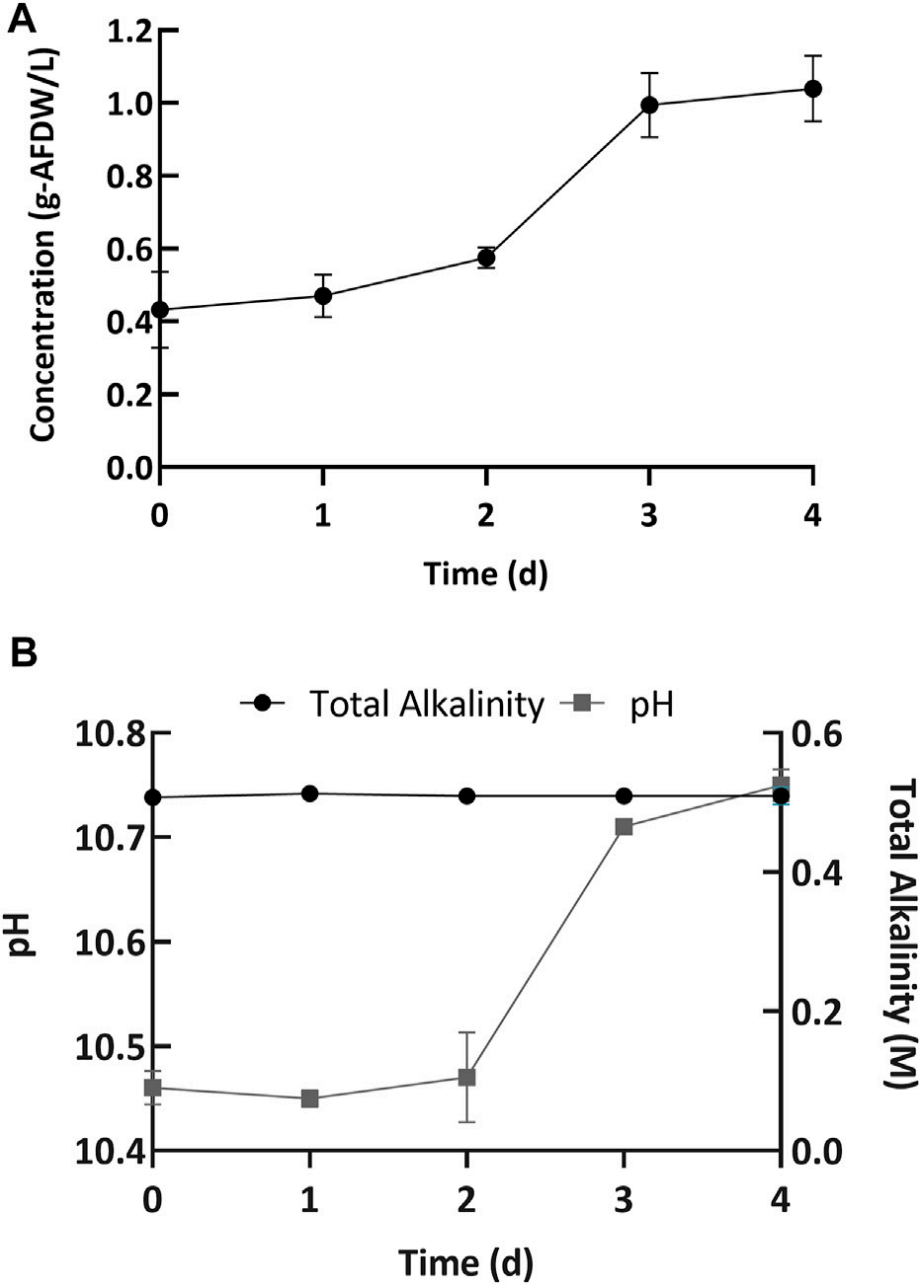
**(A)** Increase in the biomass concentration and **(B)** change in pH (left *y*-axis) and total alkalinity (right *y*-axis) over the incubation period. Error bars represent the standard deviation of the triplicate samples for each time point.

Next, we performed cultivation experiments to evaluate the biomass growth and nutrient uptake at high pH. The microbial consortium was grown in Erlenmeyer flasks (15 flasks) with a light:dark cycle of 16:8 h, with 4 days between harvests. Every day, three flasks were removed from the experiment and used for analysis. The consortium used in this study mainly consisted of (>90% relative DNA sequence abundance) the filamentous cyanobacterium *Candidatus* “Phormidium alkaliphum” (Sharp et al., 2017; Ataeian et al., 2019; Ataeian et al., 2021; Ataeian et al., 2022). This cyanobacterial consortium was inoculated at an initial concentration of 0.43 ± 0.10 g/L AFDW (Day 0, Figure 3A). Initially the alkalinity was 0.5 ± 0.003 M, and the pH was 10.46 ± 0.02 (Day 0, Figure 3B). A 4-day incubation period was chosen because the biomass growth plateaued after 4 days of incubation in previous experiments, presumably due to nitrogen sources being fully depleted (Ataeian et al., 2019). The cultures exhibited an initial lag phase followed by growth (Figure 3A). A lag phase is common for many bacteria and algae, including the green algae *Desmodesmus sp. F2* (Huang et al., 2012). Overall, cultures grew well without any apparent growth inhibition to a final biomass concentration of 1.04 ± 0.12 g/L AFDW. In parallel, the pH increased to 10.69 ± 0.1 (Day 4, Figures 3A,B). The corresponding volumetric biomass productivity (0.15 gL^−1^d^−1^ AFDW, estimated using Eq. 3) was higher than previously reported (0.048 gL^−1^d^−1^ AFDW) (Vadlamani et al., 2017). The improvement in biomass productivity was likely due to a higher initial inoculum concentration and higher light intensities used in this report.

#### Nutrient analysis of supernatant and biomass

**FIGURE 4:**
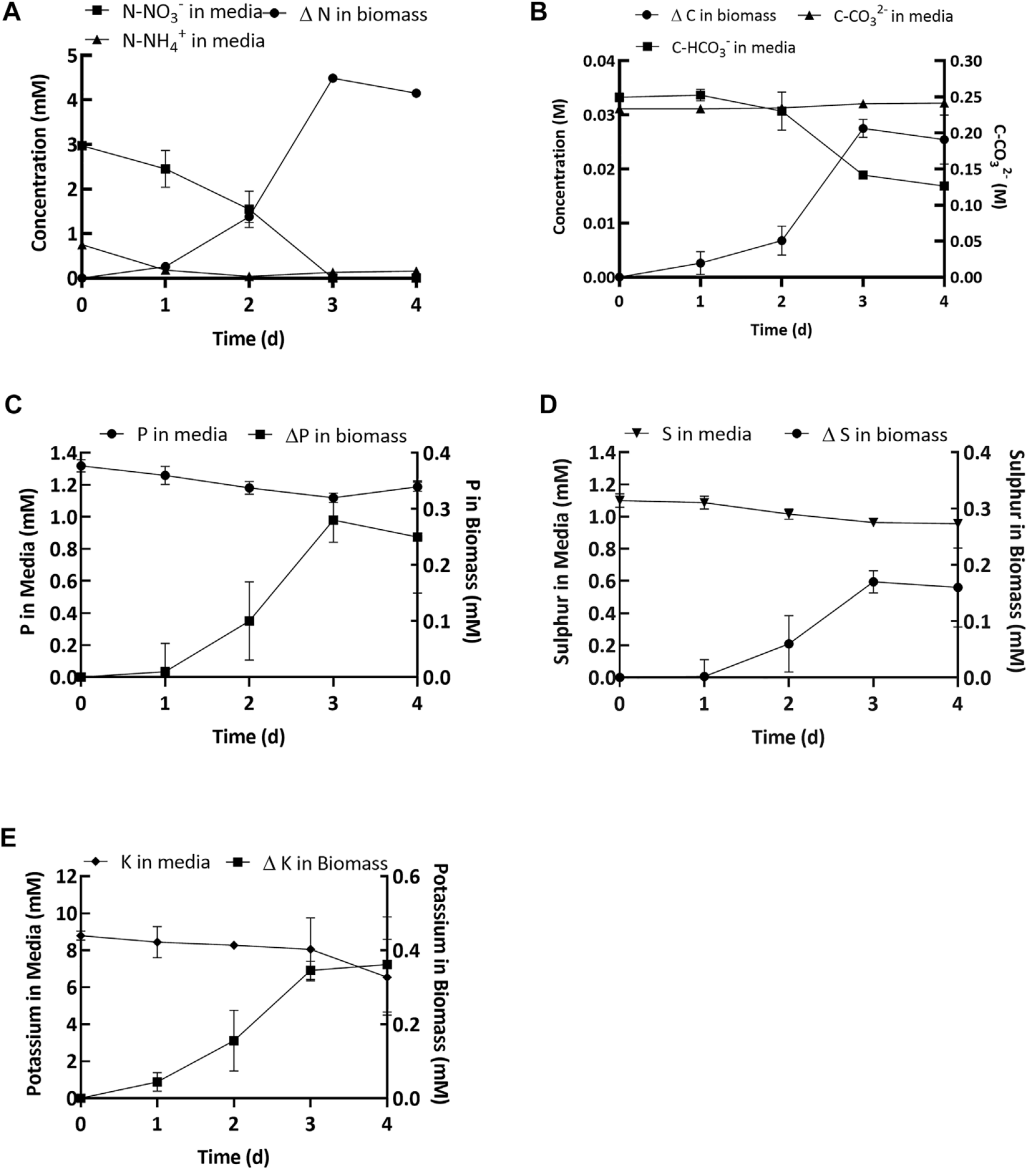
**(A)** Soluble nitrogen (NH_4_^+^ and NO_3_^−^) depleted in the media and change in biomass. **(B)** Bicarbonate (squares, left *y*-axis) and carbonate (circles, left *y*-axis) concentration in the media and change in carbon in the biomass (mM) (triangles, right *y*-axis). **(C**–**E)** Concentration of elements (P, S and K) in the spent media and change in the biomass. Values shown in the graphs are averages based on three replicates and error bars represent the standard deviation of the triplicate samples for each time point.

The estimated bicarbonate depletion observed at the end of the growth period was 16.4 ± 1.4 mM (Figure 4B). Simultaneously, an increase in carbonate concentration was also observed (8.1 ± 0.18 mM, Figure 4B). Since for every 1 mol of carbon fixed, 2 mol of bicarbonate are converted and 1 mol of carbonate is produced (Ataeian et al., 2019), the remainder of the bicarbonate decrease (8.4 ± 1.4 mM) was attributed to uptake by the cyanobacterial consortium. However, the net increase in organic carbon in the biomass (estimated from CHN analysis and gains in the ash free dry biomass concentration) was 22.9 ± 0.72 mM (Figure 4B). Thus, the net increase in organic carbon content was more than twice as much as could be sourced from the bicarbonate added to the medium. Additional bicarbonate was most likely added to the medium by spontaneous air-capture of CO_2_ during cultivation (Vadlamani et al., 2017; Ataeian et al., 2019; Vadlamani et al., 2019).

Uptake and depletion of nitrogen, phosphorus, sulfur, and potassium were analyzed along with carbon (*See* Figure 4). Figure 4A shows that the initial NO_3_^−^ concentration was 2.97 ± 0.07 and the initial NH_4_^+^ concentration was 0.75 ± 0.04 mM. By the end of the growth period the nitrogen was almost fully depleted with only 0.16 ± 0.03 mM of nitrate remaining in the media (Day 4, Figure 4A). Concomitantly, the amount of organic nitrogen in the biomass increased, equivalent to an uptake of 4.15 ± 0.03 mM (Day 4, Figure 4A). Although there was no significant increase in biomass during the lag phase (Figure 4A), 50% of the nitrogen supplied was consumed during this period (Figure 4A). The decoupling of nutrient uptake and biomass growth, known as luxurious uptake, is a well-documented phenomenon occurring in cyanobacteria and microalgae. *See* for example, Huang et al. (2012). The initial phosphorus concentration in the growth medium was 1.32 ± 0.04 mM (Day 0, Figure 4C). By the end of day 4, the final concentration of phosphorus in the media was 1.18 ± 0.03 mM (Figure 4D), which means nearly 0.14 ± 0.01 mM (Day 4, Figure 4C) of the phosphorus was depleted in the growth medium. This indicated that nearly 90% of the added phosphorus was left unused in the growth medium. Simultaneously, the concentration of phosphorus in the biomass increased by 0.25 ± 0.10 mM at the end of day 4 (Figure 4D). On day 0 the sulfur concentration in the growth medium was 1.1 ± 0.04 mM (Figure 4D) and by day 4 the final concentration was 0.95 ± 0.02 mM (Figure 4D), which means 86% of the initial sulfur added to the medium remained unused. This result was supported by the estimated uptake of sulfur in the biomass after 4 days, which was 0.16 ± 0.07 mM. Finally, the initial potassium concentration in the media was 8.79 ± 0.25 mM (Day 0, Figure 4E) and by day 3 the concentration was 8.05 ± 1.70 mM (Day 3, Figure 4E). The potassium concentration on day 4 was not reported because it had a high margin of error. The concentration of potassium in the biomass increased by 0.36 ± 0.13 mM over 4 days (Day 4, Figure 4E).

ICP-MS measurements showed that the soluble fractions of Mg, Ca, and Fe in the fresh medium were 0.30, 0.32 and 0.17, respectively in the fresh medium (Table 1). A previous study showed that the concentration of these elements is also low in the alkaline Soda Lakes (Cariboo, BC) from which this microbial community was collected (Zorz et al., 2019). We hypothesize that the alkaliphilic microbial consortium is adapted to cope with low concentrations of Mg, Ca, Co, and Fe, for example by expressing high affinity ABC transporters, producing siderophores and siderophore receptors (Chakraborty et al., 2019; Årstøl and Hohmann-Marriott, 2019). Results from Ataeian et al. (2021) revealed that the dominant species in the cyanobacteria consortium (*Candidatus* “Phormidium alkaliphilum”) used in this study, contains the genes required for iron scavenging using ABC transporters, siderophores and siderophore receptors. The type of siderophores that would be produced are classified as hydroxamates (Ataeian et al., 2021). In addition, this cyanobacterium appeared to have minimized gene content dependent on Cobalt.

The concentration of Fe, Ca, and Mg in the growth medium was depleted over the incubation period (Supplementary Figure S2). However, as Mg, Ca, and Fe precipitate at high pH, it remains unknown if these elements were assimilated by cells or if the precipitates were trapped in the extracellular polymeric layer surrounding the cells. Consequently, the depletion rate of these three elements (Fe, Ca, and Mg) in the culture medium cannot be directly equated to assimilation.

### Elemental composition and empirical formula of alkaline biomass

Using the experimental data obtained in this study, we estimated the elemental composition of the cyanobacterial consortium (Table 2). For comparison, we also analyzed the elemental composition of microbial mats collected from four different Soda Lakes located in the Cariboo Plateau, British Columbia, Canada: namely, Last Chance Lake (LCL-M), Probe Lake (PL-M), Deer Lake (DL-M), and Goodenough Lake (GEL-M). These mats were used as inoculum for the original enrichment of the cyanobacterial consortium (Sharp et al., 2017). Compared to the consortium, most mat samples were enriched in minerals, such as sodium, copper, manganese, and nickel. These were likely present as precipitates, concentrated by evaporation. Nitrogen and phosphorus were less abundant, likely because of a lower contribution of microbial cells to the mat biomass. Overall, the elemental composition obtained for both the cyanobacterial consortium and the microbial mats collected from the Soda Lakes were still comparable to previously reported pure cultures (Campanella et al., 1998; Volkman and Brown, 2006; Silva et al., 2015; Tibbetts et al., 2015).

**TABLE 2:** Comparison between elements in cultivated cyanobacteria consortium, microbial mats from soda lakes (Cariboo Plateau, British Columbia) and literature values.

Elements	Cyanobacterial consortium cultivated in lab environment		Microbial mats collected from soda lakes								Literature values^a^
	Day 3	Day 4	DL-M		PL-M		GEL-M		LC-M		
			2014	2017	2014	2017	2014	2017	2014	2017	
C (g/kg)	543.9 ± 2.6	503.0 ± 9.0	303.0	395.3	81.0	262.0	1288.12	528.0	412.0	364.9	175–650
H (g/kg)	72.6 ± 13.5	79.7 ± 31.2	43.8	55.7	11.2	34.2	192.6	73.4	54.1	51.0	ND
N (g/kg)	114.0 ± 17.45	85.2 ± 5.3	15.6	35.6	10.1	30.3	91.0	61.1	25.5	31.1	50–105
Ca (g/kg)	1.1 ± 0.4	1.5 ± 1.2	26.9	33.6	79.9	20.5	10.9	91.7	1.9	4.2	3–21
K (g/kg)	21.4 ± 3.6	14.9 ± 3.1	6.2	8.8	8.4	17.2	7.9	20.1	7.8	5.8	6–21
Mg (g/kg)	5.1 ± 0.1	4.2 ± 0.6	74.5	89.8	75.8	40.9	31.5	322.3	5.0	10.3	1–37
Na (g/kg)	63.7 ± 20.1	91.5 ± 10.1	251.4	110.4	178.1	135.3	129.6	281.8	71.6	51.9	7–321
P (g/kg)	13.3 ± 5.7	9.7 ± 3.1	1.4	4.5	6.3	17.6	3.0	11.0	1.0	2.3	0.9–30
S (g/kg)	8.1 ± 0.9	6.5 ± 1.2	5.7	9.3	36.1	10.7	81.4	18.8	21.6	9.2	4–14
Cu (mg/kg)	72.2 ± 0.4	14.7 ± 4.8	90.7	22.6	343.9	39.3	7.1	38.5	19.4	25.4	1–650
Mn (mg/kg)	88.2 ± 20.2	125.3 ± 55.3	313.9	385.1	1873.9	389.2	98.1	765.6	46.5	121.6	17–592
Zn (mg/kg)	49.5 ± 14.0	41.6 ± 9.2	51.7	50.1	212.6	51.6	16.3	75.5	14.8	30.3	2–64
Ni (mg/kg)	7.0 ± 5.7	7.1 ± 3.2	26.3	38.9	180.2	49.4	8.3	60.5	7.9	18.1	1–3
Co (mg/kg)	2.9 ± 0.9	5.9 ± 4.2	8.4	10.0	69.0	14.5	2.3	16.7	1.8	4.4	ND
Fe (mg/kg)	2.3 ± 0.5	2.9 ± 1.8	ND	ND	ND	ND	ND	ND	ND	ND	83–7,000

Deer Lake Microbial Mat (DL-M), Probe Lake Microbial Mat (PL-M), Goodenough Lake Microbial Mat (GEL-M) and Last Chance Lake Microbial Mat (LC-M). ND is no data.

a From Silva et al. (2015), Tibbetts et al. (2015), Campanella et al. (1998), Volkman and Brown (2006).

**FIGURE 5:**
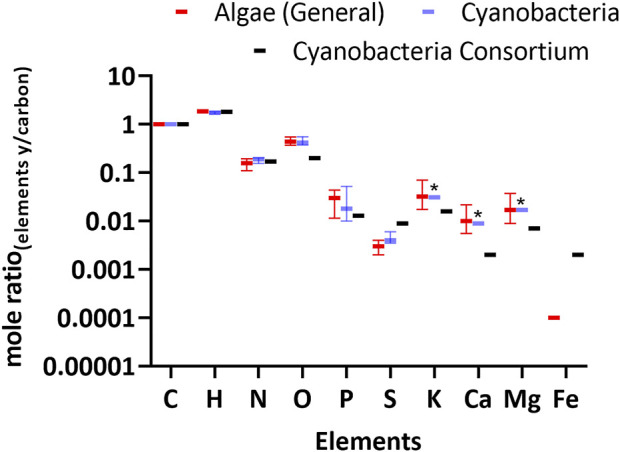
Biomass elemental composition of the cyanobacterial consortium relative to carbon. For comparison, literature values for Algae (Eukaryotes) and Cyanobacteria are shown. *See* Supplementary Table S2 for tabulated values and literature references. Error bars represent the interquartile range and solid lines represent the median. Asterisk represents the elements in the literature where only a single data point was collected for *M. aeruginosa.*

We used the elemental composition reported in Table 2 to calculate the empirical formula for the cyanobacterial consortium and microbial mats. The formula for the cyanobacterial consortium was CH_1.81_N_0.17_O_0.20_P_0.013_S_0.09;_ results for all empirical formulas are shown in Supplementary Table S1. Although this formula pertains to a microbial consortium, the stoichiometry of C to HNOPS was similar to cyanobacteria grown in pure culture (Figure 5 and Supplementary Table S2). With regards to CHNO, all forms of cellular biomass are very close, but many (eukaryotic) micro-algae have up to four times lower nitrogen content. This difference can be explained by the higher protein content of cyanobacteria.

Interestingly, the consortium’s Ca and Mg was low compared to the values previously reported for other cyanobacteria such as *M. aeruginosa*. This may be due to the consortium originating from soda lakes with a pH >10 where the solubility of Ca and Mg is low. Therefore, it may be naturally adapted to use less Ca and Mg. The iron content in the consortium was up to five times higher than for most (eukaryotic) micro-algae, but it remains unknown if this Fe was cellular or was present in the extracellular polymeric matrix.

### Regrowth of the cyanobacterial consortium in spent medium

One way to reduce the costs and improve the sustainability of algal cultivation is reusing the spent cultivation medium (Yang et al., 2011; Farooq et al., 2015; Lu et al., 2020). Depending on the culture and growth conditions, this may or may not be possible (Loftus and Johnson, 2017; Lu et al., 2020). In some cases, the reuse of spent cultivation medium has caused cultures to crash or suffer, while spent medium has also promoted growth (Lu et al., 2020).

Figure 4 shows that the inorganic carbon and nitrogen provided in the growth medium were significantly depleted. Therefore, to enable reuse of spent medium, inorganic carbon and nitrogen need to be supplemented. As more than 80% of the P, S and K remained unused (Figure 4), these nutrients would only need to be supplemented less than once every five growth cycles.

To investigate the consequences of reusing spent cultivation medium for growth, the cyanobacterial consortium was inoculated into freshly prepared media in a 12 L carboy (working volume = 10 L) at a pH of 10.5 and alkalinity of 500 mM. After 6 days the biomass was harvested by centrifugation. In the (unsterilized) spent medium, the nitrogen concentration was restored to 4 mM of combined nitrate and ammonium and the bicarbonate concentration was restored by sparging with CO_2_. The spent medium was not sterilized to mimic an actual commercial scale process more closely, where the high energy needs of sterilization would compromise both sustainability and economics. Part of the harvested biomass was added back to start the next growth cycle. In total, four growth cycles were carried out like this in triplicate (three carboy’s) using spent medium over a period of 24 days.

#### Biomass growth

**FIGURE 6:**
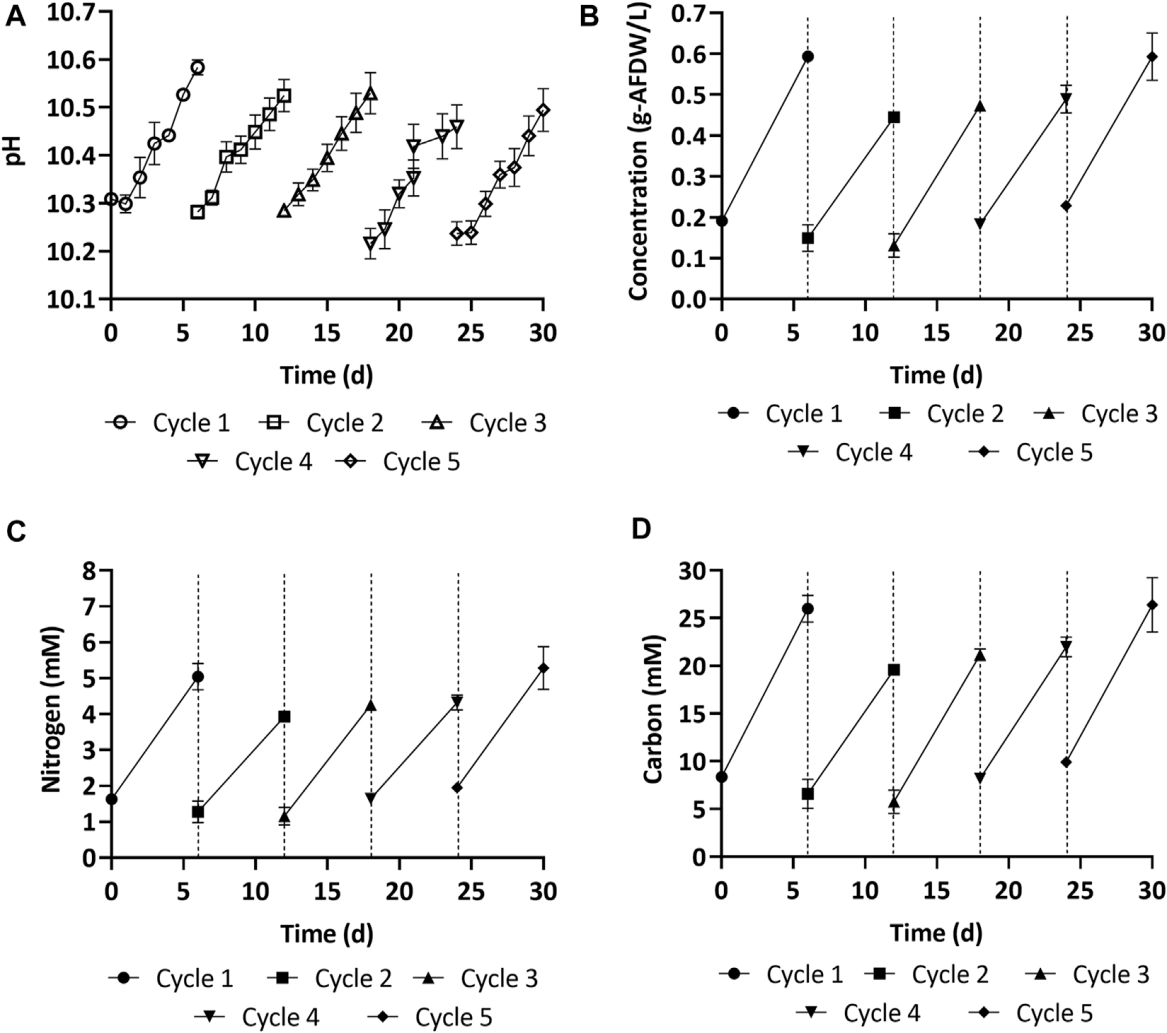
**(A**,**B)** Biomass growth and pH overtime in the fresh (cycle 1) and spent medium (cycle 2–5). **(C**,**D)** Nitrogen and carbon accumulated in the biomass overtime in the fresh (cycle 1) and spent medium (cycle 2–5). Error bars represent the standard deviation of the triplicate samples for each time point. The dashed vertical lines represent the transition between one growth cycle and the next.

Figure 6 shows the biomass growth in experiments with freshly prepared medium (cycle 1) and spent medium (cycles 2–5). The average biomass productivity (67.1 ± 0.4 mg-AFDW L^−1^d^−1^) in 12 L carboys during cycle 1 was lower than in 0.5 L Erlenmeyer flasks (150 ± 20 mg-AFDW L^−1^ d^−1^), even though the same growth medium was used (Section 3.2). This decrease in productivity was likely caused by reduced light penetration due to the larger cultivation volume (Supplementary Figure S3). The width of the 12 L carboy was 10 inches, compared to four inches for the Erlenmeyers. With spent medium in cycle 2, both the biomass concentration (0.45 g-AFDW L^−1^, *see* Figure 6B) and the estimated productivity (48.2 ± 5.7 mg-AFDW L^−1^d^−1^) decreased by 25% compared to with freshly prepared medium in cycle 1. In cultivation cycles 3 and 4, the biomass concentration and productivity recovered (Figure 6B). Nevertheless, it was still 20% lower than with fresh medium. In the final growth cycle, the biomass concentration (0.59 g-AFDW L^−1^) and productivity (60.8 ± 8.0 mg-AFDW L^−1^d^−1^) were comparable to fresh medium (*p* > 0.05, ANOVA single factor) (Figure 6B). This showed that the consortium acclimatized to the spent media. All cycles (1–5) displayed a consistent relationship between growth and pH (Figure 6A).

#### Biomass and supernatant composition

Carbon uptake, measured as AFDW, was consistent with the carbon depletion in the media. Further, the carbon to nitrogen ratio across all cycles was 1:0.2, consistent with the carbon to nitrogen ratio reported above (Figures 6C,D). The carbohydrate, protein, and ash content of the harvested biomass at the end of each cycle is reported in Table 3. The most significant change was observed in the ash content, which decreased from 21% at the end of the first cycle to 12% at the end of the last cycle. This may be explained by decreasing amounts of precipitated minerals in the media after each cycle, as part of these minerals get removed when biomass is harvested. Carbohydrates and protein content only displayed minor variations, with carbohydrates varying in the range 9–12%, and proteins fluctuating around 56–68%. This was consistent with the stable carbon to nitrogen ratio that was reported above for all cycles. These results suggest that reusing spent media did not have a significant influence on the biochemical composition of the cyanobacterial consortium.

**TABLE 3:** Biochemical composition (carbohydrates, protein, and ash) of the cyanobacterial consortium over five cycles of reusing spent medium.

Cycle	Carbohydrate (%)	Protein (%)	Ash (%)
1	10.15 ± 0.41	56.97 ± 6.19	21.19 ± 0.79
2	9.60 ± 1.06	63.35 ± 5.16	16.63 ± 0.44
3	10.58 ± 0.45	64.52 ± 1.16	15.05 ± 0.81
4	11.07 ± 0.69	64.11 ± 4.76	15.06 ± 2.23
5	11.48 ± 0.68	67.57 ± 4.58	12.05 ± 1.17

Throughout the experiments, the concentration of sodium in the media remained stable at around 500 mM, while potassium decreased from 4.72 mM ± 0.15 to 3.80 ± 0.14 mM at the end of cycle 5 (Supplementary Figure S4). Both phosphorus and sulfate concentrations decreased drastically in cycle 1 compared to the cycles where spent medium was used (cycles 2–5). Overall, the decrease of both elements after each cycle declined. For example, in cycle 2 phosphorus decreased by 17%, but decreased by only 8% in cycle 3 (Supplementary Figure S4). The phenomenon that could explain this is called luxurious uptake (Solovchenko et al., 2019). This is when microorganisms such as cyanobacteria, which grow typically in low phosphorus and sulfur environments, assimilate more than they need of any nutrient and store it. It is possible that at first, a large amount of both elements was uptaken and stored, but over time since so much was already assimilated the consortium needed less of these elements.

Previous studies using low-sodium (<20 mM) media (Mokashi et al., 2016; Barbera et al., 2018) have reported a loss of productivity due to evaporation, resulting in an increase in salinity (Discart et al., 2014; Church et al., 2017; Lu et al., 2019; Lu et al., 2020). This was not an issue for the cyanobacterial consortium used here, obtained from alkaline soda lakes, and grown in high alkalinity medium (0.5 mol L^−1^ NaHCO_3_), as shown by a complete recovery of productivity after five growth cycles. It is conceivable that the initial reduction in productivity was caused by the accumulation of organic compounds, as previously observed (Rodolfi et al., 2003; Discart et al., 2014; Depraetere et al., 2015; Lu et al., 2019; Lu et al., 2020). Indeed, the spent medium had a yellow/greenish colour, likely associated with remains of cell lysis during harvesting by centrifugation, also observed previously (Rodolfi et al., 2003; Singh and Patidar, 2018). Some of thse coloured compounds might have directly inhibited regrowth but could also simply have reduced light penetration, resulting in lower growth. In either case, if the accumulation of organic compounds was the cause of the initial loss of activity, it is likely that the heterotrophic bacteria, that were also part of the cyanobacterial consortium, started to consume these organic compounds and so facilitated the acclimatization of the culture. Many of these heterotrophs can grow on cyanobacterial metabolites and components such as cell walls, proteins, lipids and fatty acids (Ataeian et al., 2022).

## Conclusion

Growth of cyanobacteria can benefit from high pH and alkalinity by improving carbon delivery. The low solubility of iron and cobalt was shown to potentially limit growth in alkaline media. A comprehensive empirical formula was determined for an alkaliphilic cyanobacterial consortium. Reuse of spent cultivation medium was successfully demonstrated. Future research should focus on determining the practical limits for medium reuse, either by establishing a maximum number of cycles or a bleeding rate. In-depth biochemical analysis of the spent medium could help identify potential inhibitors and their tolerable concentrations. This research provides a way of improving the economics and reducing the environmental footprint of cyanobacterial cultivation, with applications in production of phycocyanin, nutraceuticals, food, and animal feed.

## Data Availability

The raw data supporting the conclusions of this article will be made available by the authors, without undue reservation.
