# Supplementary material for: Nutrient management and medium reuse for cultivation of a cyanobacterial consortium at high pH and alkalinity
Source: Front Bioeng Biotechnol. 2022 Aug 11;10:942771. doi: 10.3389/fbioe.2022.942771 (PMC9402938; doi:10.3389/fbioe.2022.942771)
Supplement: Supplementary file 1 [file Table1.DOCX]

**Supplementary Material**

$\text{Ash free dry weight} (AFDW) = ( 1 - \text{Ash content)}\times\text{Wt. of dry biomass}$ (Eq. 1)

$Biomass concentration = \frac{AFDW}{\text{Total culture volume}}$ (Eq. 2)

$Biomass productivity = \frac{{Biomass conc.}_{(f)} - {Biomass conc.}_{(i)}}{T_{(f)}-T_{(i)}}$ (Eq. 3)

Where, Biomass conc. *_(f)_* and biomass conc. *_(i)_* represent the final and initial biomass concentration values on days *T_f_* and *T_i_*, respectively.

$Uptake rate (\frac{mM}{d})=\frac{X_{f} \left( mM \right)-X_{0} (mM)}{T_{f}-T_{0}}$ (Eq. 4)

Where, X*_(f)_* and biomass X *_(0)_* represent the final and initial elemental concentration values in the biomass on days *T_f_* and *T_0_*, respectively.


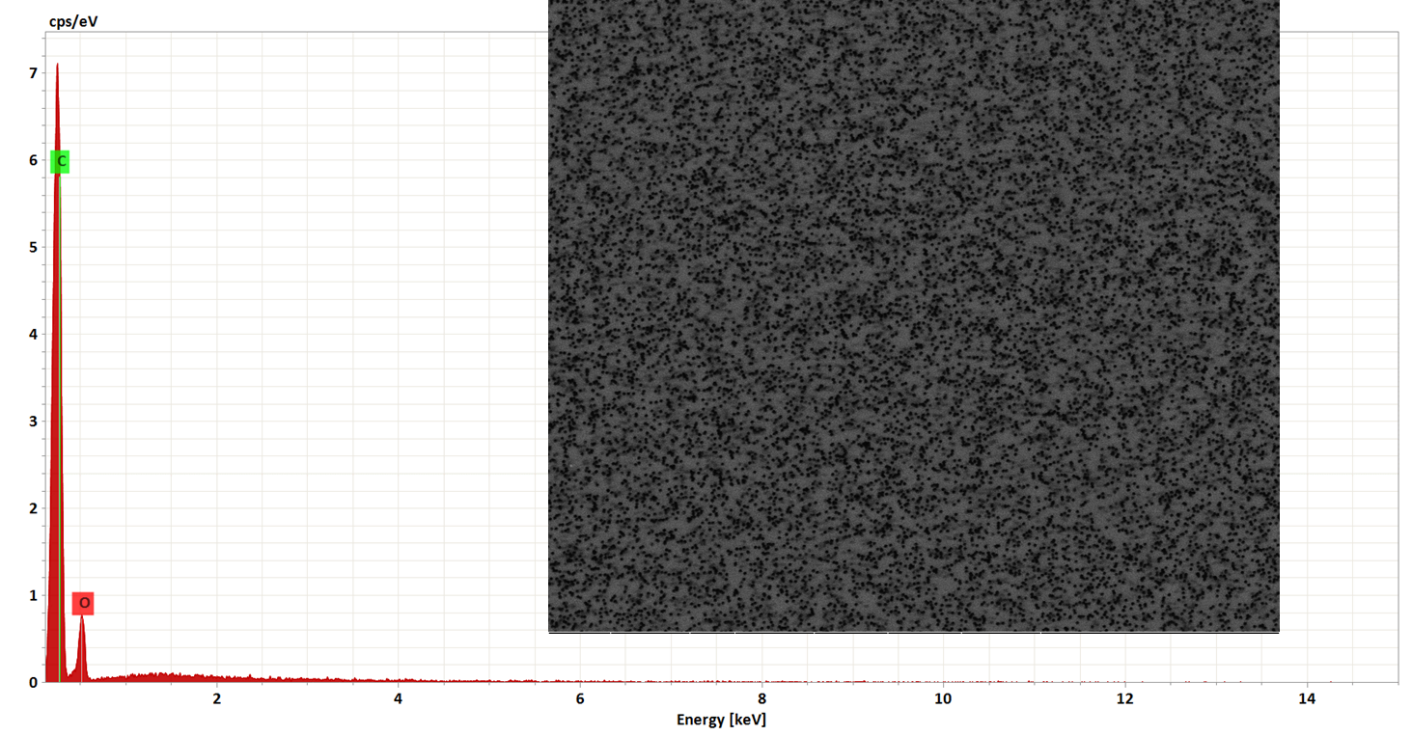


**Supplementary Figure. 1:** SEM image and EDS spectrum of filter paper (blank).

**Supplementary Figure. 2:** Concentration of calcium (a), magnesium (b) and iron in the fresh media over four days of growth. Error bars represent the standard deviation of the triplicate samples for each time point.

| **Elements** | **Cyanobacterial consortium cultivated in lab environment** | **Microbial mats collected from lakes** | | | | | | | |
| --- | --- | --- | --- | --- | --- | --- | --- | --- | --- |
|  |  | **DL-M** | | **LCL-M** | | **PL-M** | | **GEL-M** | |
|  | **Day 4** | **2014** | **2017** | **2014** | **2017** | **2014** | **2017** | **2014** | **2017** |
| **C** | 1 | 1 | 1 | 1 | 1 | 1 | 1 | 1 | 1 |
| **H** | 1.81 | 1.734653 | 1.689564 | 1.546359 | 1.67713 | 1.653333 | 1.566933 | 1.794246 | 1.668383 |
| **N** | 0.17 | 0.04413 | 0.077094 | 0.052084 | 0.07303 | 0.106667 | 0.09904 | 0.060531 | 0.099161 |
| **O** | 0.20 | ND | ND | ND | ND | ND | ND | ND | ND |
| **P** | 1.30E-02 | 1.18E-03 | 2.92E-03 | 3.99E-04 | 1.81E-03 | 1.66E-02 | 2.11E-02 | 1.60E-04 | 4.46E-03 |
| **S** | 9.00E-03 | 4.67E-03 | 5.89E-03 | 8.77E-03 | 6.92E-03 | 9.29E-02 | 1.24E-02 | 4.22E-03 | 7.37E-03 |
| **Mg** | 6.67E-03 | 8.19E-02 | 7.58E-02 | 2.71E-03 | 1.03E-02 | 2.60E-01 | 6.34E-02 | 2.18E-03 | 1.68E-01 |
| **Ca** | 1.97E-03 | 1.78E-02 | 1.70E-02 | 6.06E-04 | 2.54E-03 | 2.60E-01 | 1.91E-02 | 4.53E-04 | 2.87E-02 |
| **K** | 1.63E-02 | 4.20E-03 | 4.56E-03 | 2.59E-03 | 3.61E-03 | 1.77E-02 | 1.64E-02 | 3.36E-04 | 6.47E-03 |
| **Na** | 0.11 | 2.89E-01 | 9.71E-02 | 4.04E-02 | 5.45E-02 | 6.37E-01 | 2.19E-01 | 9.35E-03 | 1.54E-01 |
| **Fe** | 1.96E-03 | 5.49E-03 | 6.03E-03 | 6.46E-04 | 3.09E-03 | 1.63E-01 | 1.37E-02 | 1.08E-04 | 6.63E-03 |
| **Mn** | 6.00E-05 | 1.51E-04 | 1.42E-04 | 1.10E-05 | 5.33E-05 | 2.80E-03 | 2.63E-04 | 2.96E-06 | 1.75E-04 |
| **Co** | 2.12E-06 | 3.77E-06 | 3.43E-06 | 4.04E-07 | 1.80E-06 | 9.62E-05 | 9.13E-06 | 6.57E-08 | 3.55E-06 |
| **Ni** | 5.66E-06 | 1.16E-05 | 1.31E-05 | 1.70E-06 | 7.28E-06 | 2.47E-04 | 3.07E-05 | 2.30E-07 | 1.26E-05 |
| **Cu** | 1.01E-05 | 3.80E-05 | 7.27E-06 | 4.00E-06 | 9.74E-06 | 4.49E-04 | 2.32E-05 | 1.86E-07 | 7.67E-06 |
| **Zn** | 2.76E-05 | 2.07E-05 | 1.54E-05 | 2.91E-06 | 1.11E-05 | 2.65E-04 | 2.91E-05 | 4.09E-07 | 1.44E-05 |
| **B** | 2.52E-05 | 4.51E-05 | 3.65E-05 | 1.15E-04 | 4.09E-04 | 3.83E-04 | 1.13E-04 | 5.12E-06 | 2.31E-04 |
| Deer Lake Microbial Mat (DL-M), Probe Lake Microbial Mat (PL-M), Goodenough Lake Microbial Mat (GEL-M) and  Last Chance Lake Microbial Mat (LC-M)  ND is No Data | | | | | | | | | |
|  |  |  |  |  |  |  |  |  |  |

**Supplementary Table 1**: Shows empirical formula of the cyanobacterial consortium cultivated in controlled laboratory environment on day and the media microbial mats collected in 2014 and 2017 from four soda lakes in the Cariboo Plateau, British Columbia, Canada.

**Supplementary Table 2**: Empirical formula for the cyanobacterial consortium's elemental composition compared to previously reported data for cyanobacteria and microalgae. The values for some elements are not shown for lack of literature data. These are Mn (xx), Co (xx), Ni, Cu (x), Zn (z) and B (x).

|  | C | H | N | O | P | S | K | Ca | Mg | Fe | Paper |
| --- | --- | --- | --- | --- | --- | --- | --- | --- | --- | --- | --- |
| Cyanobacteria |  |  |  |  |  |  |  |  |  |  |  |
| Cyanobacterial Consortium | 1 | 1.81 | 0.17 | 0.20 | 0.013 | 0.009 | 0.016 | 0.002 | 0.007 | 0.002 | This Paper |
| Chroococcus sp. | 1 | 1.55 | 0.09 | 0.25 | ND | ND | ND | ND | ND | ND | Prajapati et al. (2014) |
| Anabaena variabilis (airlift) | 1 | 1.71 | 0.19 | 0.42 | ND | ND | ND | ND | ND | ND | Fontes et al. (1989), Table 2 |
| Anabaena variabilis (paddlewheel) | 1 | 1.84 | 0.19 | 0.39 | ND | ND | ND | ND | ND | ND | Fontes et al. (1989), Table 2 |
| S. platensis | 1 | 1.77 | 0.19 | 0.56 | ND | 0.004 | ND | ND | ND | ND | Jena et al. (2011), Table 1 |
| Spirulina platensis strain (UTEX 1926) | 1 | 1.75 | 0.14 | 0.67 | ND | 0.006 | ND | ND | ND | ND | Arata et al. (2013), Table 1 |
| Spirulina platensis strain 8005 | 1 | 1.64 | 0.17 | 0.55 | ND | 0.006 | ND | ND | ND | ND | Arata et al. (2013) and Cornet et al. (1992) |
| Synechocystis sp. PCC6803 | 1 | 1.62 | 0.22 | 0.4 | 0.01 | ND | ND | ND | ND | ND | Kim et al. (2011) |
| Microcystis aeruginosa | 1 | 1.785 | 0.207 | 0.487 | 0.052 | 0.003 | 0.031 | 0.009 | 0.017 | ND | Picardo et al 2013 |
| Spirulina platensis | 1 | 1.862 | 0.2 | 0.354 | 0.018 | 0.004 | ND | ND | ND | ND | Picardo et al 2013 |
| Eukaryotic Algae |  |  |  |  |  |  |  |  |  |  |  |
| Algae (General) | 1 | 2.481 | 0.151 | 1.038 | 0.009 | ND | ND | ND | ND | ND | Popovic (2019), Table 1 |
| Chlamydomonas | 1 | 1.65 | 0.12 | 0.39 | ND | ND | ND | ND | ND | ND | Popovic (2019), Table 1 |
| Chlorella | 1 | 1.719 | 0.175 | 0.404 | 0.011 | ND | ND | ND | ND | ND | Popovic (2019), Table 1 |
| Chlorella a sp. MP-1 | 1 | 1.793 | 0.121 | 0.608 | ND | ND | ND | ND | ND | ND | Popovic (2019), Table 1 |
| Chlorella minutissima | 1 | 1.714 | 0.143 | 0.286 | ND | ND | ND | ND | ND | ND | Popovic (2019), Table 1 |
| Chlorella pyrenoidosa | 1 | 1.625 | 0.125 | 0.25 | ND | ND | ND | ND | ND | ND | Popovic (2019), Table 1 |
| Chlorella vulgaris | 1 | 1.667 | 0.111 | 0.222 | ND | ND | ND | ND | ND | ND | Popovic (2019), Table 1 |
| Chlorella sp. ATCC 7516 (medium 5) | 1 | 1.76 | 0.09 | 0.35 | ND | ND | ND | ND | ND | ND | Popovic (2019), Table 1 |
| Chlorella Spain sp. ATCC 7516 (medium S) | 1 | 1.78 | 0.12 | 0.36 | ND | ND | ND | ND | ND | ND | Popovic (2019), Table 1 |
| Rocan 1 | 1 | 1.4 | 0.04 | 0.5 | ND | ND | ND | ND | ND | ND | Popovic (2019), Table 1 |
| Rocan BUV 2 | 1 | 1.56 | 0.05 | 0.59 | ND | ND | ND | ND | ND | ND | Popovic (2019), Table 1 |
| Scenedesmus obtusiusculus | 1 | 1.64 | 0.11 | 0.44 | ND | ND | ND | ND | ND | ND | Popovic (2019), Table 1 |
| Selenastrum capricornutum | 1 | 1.6 | 0.08 | 0.43 | ND | ND | ND | ND | ND | ND | Popovic (2019), Table 1 |
| D. tertiolecta | 1 | 1.65 | 0.04 | 0.84 | ND | ND | ND | ND | ND | ND | Chen et al. (2017) |
| Aphanothece microscopica Nägeli | 1 | 1.999 | 0.184 | 0.518 | 0.04 | 0.005 | 0.048 | 0.014 | 0.026 | ND | Picardo et al. (2013) |
| Botryococcus braunii | 1 | 1.838 | 0.069 | 0.233 | 0.007 | 0.001 | ND | ND | ND | ND | Picardo et al. (2013) |
| Chaetoceros sp. | 1 | 1.923 | 0.193 | 0.431 | 0.035 | 0.004 | 0.032 | 0.009 | 0.017 | ND | Picardo et al. (2013) |
| Chaetoceros calcitrans | 1 | 1.926 | 0.17 | 0.602 | 0.07 | 0.003 | 0.079 | 0.023 | 0.042 | 0.0001 | Picardo et al. (2013) |
| Chaetoceros calcitrans f. pumilus | 1 | 1.777 | 0.17 | 0.426 | 0.027 | 0.002 | ND | ND | ND | ND | Picardo et al. (2013) |
| Chaetoceros cf. wighamii | 1 | 1.732 | 0.255 | 0.478 | 0.04 | 0.003 | ND | ND | ND | ND | Picardo et al. (2013) |
| Chaetoceros gracilis | 1 | 1.491 | 0.298 | 0.513 | 0.069 | 0.001 | ND | ND | ND | ND | Picardo et al. (2013) |
| Chaetoceros muelleri | 1 | 1.777 | 0.217 | 0.452 | 0.032 | 0.003 | ND | ND | ND | ND | Picardo et al. (2013) |
| Chlamydomonas reinhardtii | 1 | 1.909 | 0.162 | 0.36 | 0.011 | 0.003 | ND | ND | ND | ND | Picardo et al. (2013) |
| Chlamydomonas sp. | 1 | 1.982 | 0.048 | 0.544 | 0.001 | 0.001 | ND | ND | ND | ND | Picardo et al. (2013) |
| Chlorela pyrenoidosa | 1 | 1.934 | 0.23 | 0.473 | 0.017 | 0.005 | ND | ND | ND | ND | Picardo et al. (2013) |
| Chlorella sp. | 1 | 1.861 | 0.182 | 0.339 | 0.016 | 0.003 | ND | ND | ND | ND | Picardo et al. (2013) |
| Chlorella minutissima | 1 | 1.943 | 0.068 | 0.42 | 0.002 | 0.002 | ND | ND | ND | ND | Picardo et al. (2013) |
| Chlorella protothecoides | 1 | 1.914 | 0.193 | 0.383 | 0.022 | 0.004 | 0.013 | 0.004 | 0.007 | ND | Picardo et al. (2013) |
| Chlorella sorokiniana | 1 | 1.987 | 0.115 | 0.431 | 0.002 | 0.003 | ND | ND | ND | ND | Picardo et al. (2013) |
| Chlorella vulgaris | 1 | 1.995 | 0.084 | 0.525 | 0.002 | 0.002 | ND | ND | ND | ND | Picardo et al. (2013) |
| Cryptomonas sp. | 1 | 1.917 | 0.173 | 0.349 | 0.03 | 0.004 | 0.029 | 0.008 | 0.016 | ND | Picardo et al. (2013) |
| Cyclotella cryptica | 1 | 1.996 | 0.087 | 0.608 | 0.043 | 0.002 | 0.058 | 0.017 | 0.031 | 0.0001 | Picardo et al. (2013) |
| Dunaliella salina | 1 | 2.043 | 0.157 | 0.492 | 0.012 | 0.004 | 0.016 | 0.005 | 0.008 | ND | Picardo et al. (2013) |
| Dunaliella tertiolecta | 1 | 1.664 | 0.22 | 0.45 | 0.043 | 0.002 | ND | ND | ND | ND | Picardo et al. (2013) |
| Haematococcus pluvialis | 1 | 2.021 | 0.104 | 0.574 | 0.006 | 0.003 | 0.005 | 0.002 | 0.003 | ND | Picardo et al. (2013) |
| Isochrysis sp. | 1 | 1.982 | 0.141 | 0.408 | 0.032 | 0.004 | 0.044 | 0.013 | 0.023 | ND | Picardo et al. (2013) |
| Isochrysis galbana | 1 | 1.884 | 0.089 | 0.316 | 0.027 | 0.002 | 0.03 | 0.009 | 0.016 | ND | Picardo et al. (2013) |
| Isochrysis aff. galbana | 1 | 1.966 | 0.109 | 0.339 | 0.001 | 0.003 | 0.002 | ND | 0.001 | ND | Picardo et al. (2013) |
| Micromonas pusilla | 1 | 1.944 | 0.164 | 0.302 | 0.018 | 0.004 | 0.019 | 0.005 | 0.01 | ND | Picardo et al. (2013) |
| Nannochloropsis oculata | 1 | 1.76 | 0.208 | 0.377 | 0.03 | 0.003 | ND | ND | ND | ND | Picardo et al. (2013) |
| Nanochloropsis salina | 1 | 1.82 | 0.125 | 0.275 | 0.014 | 0.002 | ND | ND | ND | ND | Picardo et al. (2013) |
| Navicula incerta | 1 | 1.835 | 0.174 | 1.702 | 0.384 | 0.002 | 0.48 | 0.138 | 0.258 | 0.0005 | Picardo et al. (2013) |
| Nitzschia closterium | 1 | 1.683 | 0.233 | 0.441 | 0.042 | 0.002 | ND | ND | ND | ND | Picardo et al. (2013) |
| Nitzschia frustulum | 1 | 1.756 | 0.2 | 0.572 | 0.1 | 0.002 | 0.096 | 0.028 | 0.051 | 0.0001 | Picardo et al. (2013) |
| Nitzschia sp. | 1 | 1.786 | 0.194 | 0.485 | 0.071 | 0.003 | 0.062 | 0.018 | 0.033 | 0.0001 | Picardo et al. (2013) |
| Pavlova lutheri | 1 | 2.015 | 0.145 | 0.475 | 0.011 | 0.004 | 0.012 | 0.004 | 0.007 | ND | Picardo et al. (2013) |
| Pavlova salina | 1 | 1.661 | 0.246 | 0.441 | 0.045 | 0.002 | ND | ND | ND | ND | Picardo et al. (2013) |
| Phaeodactylum tricornutum | 1 | 1.984 | 0.157 | 0.354 | 0.018 | 0.004 | 0.024 | 0.007 | 0.013 | ND | Picardo et al. (2013) |
| Porphyridium cruentum | 1 | 1.931 | 0.165 | 0.619 | 0.037 | 0.003 | 0.029 | 0.008 | 0.016 | ND | Picardo et al. (2013) |
| Pyramimonas sp. | 1 | 2.054 | 0.163 | 0.545 | 0.022 | 0.004 | 0.029 | 0.008 | 0.016 | ND | Picardo et al. (2013) |
| Rhodomonas sp. | 1 | 1.887 | 0.218 | 0.444 | 0.047 | 0.004 | 0.04 | 0.011 | 0.021 | ND | Picardo et al. (2013) |
| Scenedesmus almeriensis | 1 | 1.953 | 0.172 | 0.44 | 0.012 | 0.004 | 0.004 | 0.001 | 0.002 | ND | Picardo et al. (2013) |
| Skeletonema costatum | 1 | 1.917 | 0.15 | 0.621 | 0.094 | 0.003 | 0.115 | 0.033 | 0.062 | 0.0001 | Picardo et al. (2013) |
| Skeletonema sp. | 1 | 1.804 | 0.199 | 0.551 | 0.092 | 0.003 | 0.093 | 0.027 | 0.05 | 0.0001 | Picardo et al. (2013) |
| Tetraselmis chuii | 1 | 1.755 | 0.202 | 0.407 | 0.031 | 0.002 | ND | ND | ND | ND | Picardo et al. (2013) |
| Tetraselmis suecica | 1 | 1.85 | 0.23 | 0.541 | 0.068 | 0.004 | 0.06 | 0.017 | 0.032 | 0.0001 | Picardo et al. (2013) |
| Thalassiosira pseudonana | 1 | 1.955 | 0.151 | 0.681 | 0.093 | 0.003 | 0.116 | 0.033 | 0.062 | 0.0001 | Picardo et al. (2013) |

ND is No Data


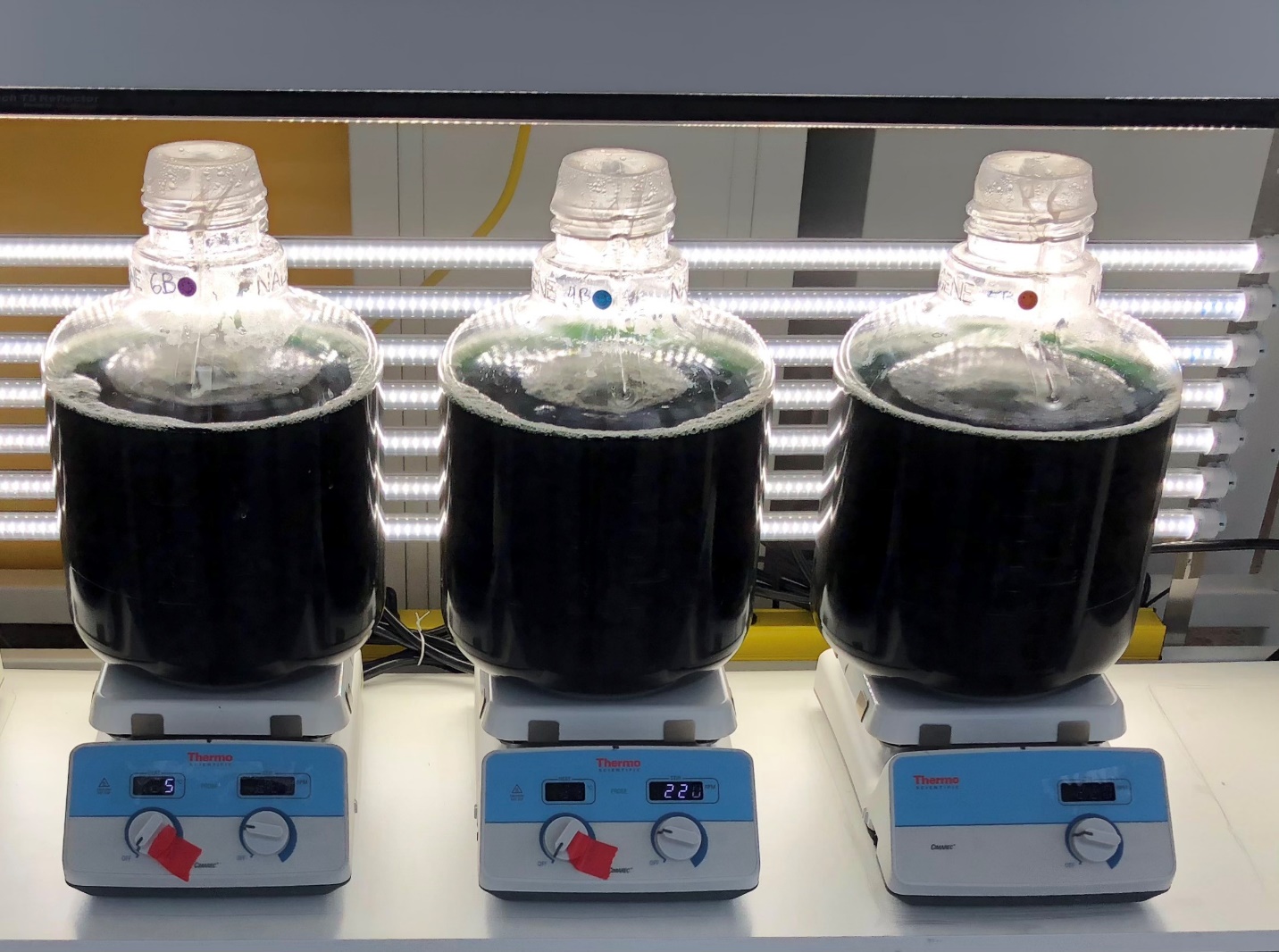


**Supplementary Figure 3:** Cyanobacterial consortium growth in a 12 L carboy with a diameter of 10 inches. For every cultivation cycle 10 L of media (fresh or spent medium) was added to the carboy with ~10 grams of culture and the consortium was cultivated for a period of 6 days for each cycle.

**Supplementary Figure 4:** (a-d) Concentration of phosphorus, sulfur, potassium and sodium in the fresh (cycle 1) and spent media (cycle 2-5) overtime. Values shown in the graphs are averages based on three replicates and error bars represent the standard deviation of the triplicate samples for each time point.
